# ULBP1 is a prognostic biomarker associated with immune suppression in breast cancer

**DOI:** 10.3389/fonc.2026.1788245

**Published:** 2026-08-26

**Authors:** Qixin Mao, Yu Zheng, Shanqing Liu, Yong Li, Yan Shen, Hao Zhuang

**Affiliations:** 1 Department of Breast Disease, Henan Breast Cancer Center, The Affiliated Cancer Hospital of Zhengzhou University & Henan Cancer Hospital, Zhengzhou, Henan, China; 2 Tianjin Medical University Cancer Institute & Hospital, National Clinical Research Center for Cancer, Tianjin’s Clinical Research Center for Cancer, Tianjin Medical University, Ministry of Education, Key Laboratory of Cancer Prevention and Therapy, Tianjin, China; 3 Tianjin Medical University Cancer Institute & Hospital, National Clinical Research Center for Cancer, Tianjin’s Clinical Research Center for Cancer, Tianjin Medical University, Key Laboratory of Breast Cancer Prevention and Therapy, Tianjin, China; 4 Department of Hepatobiliopancreatic Surgery, The Affiliated Cancer Hospital of Zhengzhou University & Henan Cancer Hospital, Zhengzhou, Henan, China

**Keywords:** breast cancer, drug sensitivity, prognosis, tumor immune microenvironment, ULBP1

## Abstract

**Background:**

Breast cancer (BC) is the most common malignant tumor among women worldwide. ULBP1 (UL16 binding protein 1), as a ligand of NKG2D, has been associated with poor prognosis in multiple cancers; however, its role in BC remains unclear.

**Methods:**

TCGA and GEO datasets (TCGA-BRCA, GSE36295, and GSE1379) were used to evaluate the expression characteristics of ULBP1 and its association with prognosis. Functional enrichment analyses were subsequently performed to identify key biological pathways. The CIBERSORT and oncoPredict algorithms were applied to assess immune infiltration features and differences in drug sensitivity, respectively. In addition, qRT-PCR, western blotting, as well as CCK-8 and Transwell assays were conducted to validate ULBP1 expression in BC cells and to examine its effects on cellular functions.

**Results:**

Analysis of public datasets revealed that ULBP1 is significantly overexpressed in BC tissues, and its high expression was independently associated with shortened overall survival. GSEA revealed that high ULBP1 expression was significantly enriched in immune-related pathways, including natural killer cell–mediated cytotoxicity. Immune infiltration analysis indicated that high ULBP1 expression was closely associated with neutrophil enrichment and the upregulation of multiple immune checkpoint genes. Drug sensitivity prediction showed that patients with high ULBP1 expression may be more sensitive to olaparib and other agents. *In vitro* experiments further confirmed that ULBP1 was highly expressed in BC cell lines, and that knockdown of ULBP1 significantly suppressed MCF-7 cell viability and reduced migratory and invasive capacities.

**Conclusion:**

ULBP1 may serve as a potential novel prognostic biomarker in BC, and targeting ULBP1 may provide a new therapeutic strategy for BC.

## 1 Introduction

Breast cancer (BC) remains the most frequently diagnosed malignant tumor among women worldwide and is also a leading cause of cancer-related mortality (1, 2). According to the most recent global cancer statistics (GLOBOCAN), BC accounts for approximately 11.6% of all newly diagnosed malignancies, imposing a substantial burden on global public health systems (3). Although the implementation of early screening programs has significantly improved survival outcomes for patients with localized BC, the prognosis of patients with advanced or metastatic disease remains unsatisfactory (4).

Currently, the clinical management of BC primarily relies on molecular subtype–guided multimodal therapeutic strategies, including surgical resection, radiotherapy, chemotherapy, endocrine therapy, and HER2-targeted therapy (5). Nevertheless, despite the overall survival (OS) benefit achieved in a subset of patients, a considerable proportion ultimately experience disease progression. This can be attributed, on the one hand, to the pronounced biological heterogeneity of BC, particularly triple-negative breast cancer (TNBC), which lacks specific therapeutic targets and is frequently characterized by high aggressiveness and recurrence rates (6). On the other hand, the efficacy of current treatments is often limited by intrinsic or acquired drug resistance, as well as severe systemic toxicity (7, 8). Therefore, there is an urgent need to further identify novel and robust biomarkers with diagnostic and therapeutic relevance, thereby providing new targets for optimizing treatment strategies and improving patient outcomes.

ULBP1 (UL16 binding protein 1) is a member of the NKG2D receptor ligand family and primarily functions by binding to the NKG2D receptor expressed on natural killer (NK) cells and certain subsets of T cells, thereby activating immune responses (9). In BC, NKG2D ligands, including ULBP1, have been frequently detected in tumor tissues by immunohistochemistry, and their expression has been reported to correlate with clinical outcomes (10, 11). Previous studies have further demonstrated that ULBP1 is highly expressed in multiple malignancies, such as glioblastoma, colorectal adenocarcinoma, and hepatocellular carcinoma, where its elevated expression is closely associated with poor prognosis (12–15). Although the functional significance of ULBP1 has been explored in several tumor types and its contribution to cancer immune evasion has been increasingly recognized, its precise role in BC remains insufficiently elucidated.

Therefore, the present study focuses on ULBP1 as the target of investigation, aiming to analyze its expression patterns in BC tissues, elucidate its regulatory effects on BC cell proliferation, migration, and invasion, and to further clarify the association between ULBP1 and tumor progression. The findings of this study are expected to provide new insights into the molecular mechanisms and immune regulation of BC and to lay a theoretical foundation for the development of novel therapeutic strategies targeting ULBP1.

## 2 Materials and methods

### 2.1 Database data

Gene expression profiles and corresponding clinical follow-up information for the BC (BRCA) cohort were obtained from The Cancer Genome Atlas (TCGA) database (https://portal.gdc.cancer.gov/). A total of 1271 samples were included in this cohort, comprising 1,104 primary tumor samples and 113 adjacent normal breast tissue samples. Among these, 1,082 tumor samples had complete survival follow-up data and were used for subsequent prognostic analyses. In addition, three independent BC–related datasets were retrieved from the Gene Expression Omnibus (GEO) database (https://www.ncbi.nlm.nih.gov/geo/): GSE36295, which included 50 samples (45 tumor samples and 5 normal samples), GSE1379, which consisted of 60 tumor samples, all with complete survival information and GSE124821, which comprised RNA-seq data from novel syngeneic BC models under treated or untreated conditions.

### 2.2 Differentially expressed genes analysis

For the TCGA dataset, differential expression analysis was performed using the DESeq2 package in R (16). For the GSE36295 dataset, differential expression analysis was conducted using the limma package in R (17). The screening criteria for differentially expressed genes (DEGs) were set as follows: an absolute log2-transformed fold change (|log2FC|) > 1 and a false discovery rate (FDR) < 0.05.

### 2.3 Functional enrichment and pathway analysis

To investigate the functional implications of the DEGs, we utilized the R package clusterProfiler (v4.8.3) (18). This involved conducting Gene Ontology (GO) enrichment across three domains—Biological Process (BP), Molecular Function (MF), and Cellular Component (CC)—alongside Kyoto Encyclopedia of Genes and Genomes (KEGG) pathway analysis. A P-value threshold of 0.05 was applied to determine statistical significance for both analyses. Additionally, Gene set enrichment analysis (GSEA) was performed to compare distinct groups, with significance defined by an adjusted P-value < 0.05.

### 2.4 Univariate cox regression analysis

Using survival information and gene expression data from the TCGA-BRCA cohort and GEO datasets, we identified prognostic markers via univariate Cox regression analysis. The survival and survminer R packages were employed for these calculations, with a P-value < 0.05 serving as the threshold to determine significant correlation with patient survival.

### 2.5 Survival analysis and prognostic evaluation

OS was estimated using the Kaplan–Meier method with the survival and survminer packages in R (19), and survival differences between groups were assessed using the log-rank test. Median- or quartile-based stratification of ULBP1 expression was used only for Kaplan–Meier visualization, external-cohort survival analysis, and exploratory group-based comparisons. For the primary prognostic analysis, ULBP1 expression was entered into Cox proportional hazards regression models as a continuous variable. To evaluate whether ULBP1 was independently associated with prognosis, multivariate Cox proportional hazards regression models were constructed after adjustment for key clinicopathological variables. Receiver operating characteristic (ROC) curves were generated using the pROC package in R (20), and the area under the curve (AUC) was calculated to evaluate the discriminatory ability of the model or individual genes. Time-dependent ROC curves were generated using the timeROC package (21, 22), and AUC values at different time points were calculated to assess the dynamic predictive performance of the prognostic model.

### 2.6 Tumor immune infiltration analysis

The CIBERSORT algorithm (23) was applied to estimate the relative proportions of 22 immune cell subtypes in tumor tissues based on a predefined signature matrix of 547 genes using a deconvolution approach. The analysis was performed with 100 permutations (perm = 100) and quantile normalization enabled (QN = TRUE) to minimize technical variation across samples. The sum of the proportions of all immune cell types in each sample was constrained to equal 1. The ESTIMATE package (24) was used to calculate stromal and immune scores for each sample based on stromal- and immune-related gene signatures, thereby quantifying the infiltration levels of stromal and immune cells within the tumor microenvironment (TME). The tumor immune dysfunction and exclusion (TIDE) score was calculated for each sample using the online TIDE tool (http://tide.dfci.harvard.edu/).

### 2.7 Drug sensitivity prediction analysis

Drug sensitivity prediction was performed using the oncoPredict package in R (25). The GDSC2_Expr expression matrix, containing 17,419 genes across 805 cell lines, and the GDSC2_Res drug response matrix, containing IC_50_ values for 198 drugs across 805 cell lines, were used as the training datasets. Based on the standardized TCGA-BRCA transcriptomic data, the calcPhenotype function was applied to predict drug-specific IC_50_ values for BC samples, thereby indirectly estimating potential differences in drug sensitivity among samples. Associations between ULBP1 expression and predicted drug sensitivity were then evaluated across the drug panel. To account for multiple comparisons, p values were adjusted across the drug panel using a false discovery rate approach. Correlation coefficients and corresponding confidence intervals were calculated to report effect sizes and uncertainty.

### 2.8 Cell culture and transfection

Four cell lines were employed in this study to represent the major molecular subtypes of BC. MCF-10A, a normal mammary epithelial cell line, was used as a non-tumorigenic control (26). MCF-7 is a hormone receptor-positive (ER+/PR+) BC cell line (27). MDA-MB-231 is a TNBC cell line representing a highly aggressive BC subtype (27). SKBR3 is a HER2-positive BC cell line (28). Together, these cell lines enable a comprehensive assessment of ULBP1 expression across distinct BC molecular subtypes, thereby improving the representativeness and generalizability of the findings.

MCF-10A cells (CL-0525, Procell) were cultured in the corresponding specialized medium (CM-0525, Procell). MDA-MB-231 cells (CL-0150, Procell) were maintained in DMEM medium (PM150270, Procell) supplemented with 10% fetal bovine serum (FBS; 164210, Procell) and 1% penicillin–streptomycin (P/S; P1400, Solarbio). MCF-7 cells (CL-0149, Procell) were cultured in MEM medium (PM150410, Procell) supplemented with 10% FBS and 1% P/S, while SKBR3 cells (CL-0211, Procell) were cultured in McCoy’s 5A medium (PM150710, Procell) supplemented with 10% FBS and 1% P/S. All cell lines were authenticated by short tandem repeat (STR) profiling to confirm their identity and were tested negative for mycoplasma contamination. All cells were maintained at 37 °C in a humidified atmosphere containing 5% CO_2_ and passaged when reaching 80–90% confluence.

MCF-7 cells were transfected with negative control siRNA (si-NC) or ULBP1-targeting siRNAs (si-ULBP1-1/2/3) using a transfection reagent kit (AQ11668, Beijing Aoqing Biotechnology Co., Ltd.) according to the manufacturer’s instructions. The siRNA sequences are provided in **Supplementary Table 1**.

For ULBP1 overexpression in MDA-MB-231 cells, cells were transfected with pcDNA3.1 negative control (OE-NC) and pcDNA3.1-ULBP1 (OE-ULBP1) plasmids using Lipofectamine 2000 reagent.

### 2.9 Cell viability assay

Following transfection with si-ULBP1 or negative control (si-NC), cells were distributed into 96-well plates at a concentration of 2,500 cells per well. Cell viability was assessed using the Cell Counting Kit-8 assay (CCK-8; C0037, Beyotime) according to the manufacturer’s instructions. Briefly, at the indicated time points up to 72 h after seeding, 10 μL of CCK-8 reagent was added to each well, followed by incubation at 37 °C for 2 h. The absorbance at 450 nm was then measured using an Infinite F50 microplate reader (Tecan) to evaluate cell viability.

### 2.10 Olaparib sensitivity assay

To experimentally validate the predicted association between ULBP1 expression and olaparib sensitivity, OE-NC and OE-ULBP1 MCF-7 cells were seeded into 96-well plates at a density of 2,500 cells per well. After cell attachment, cells were treated with olaparib at concentrations of 1, 10, or 50 μM. Cell viability was assessed at the indicated time points using the CCK-8 assay according to the manufacturer’s instructions. Briefly, 10 μL of CCK-8 reagent was added to each well and incubated at 37 °C for 2 h. The absorbance at 450 nm was measured using an Infinite F50 microplate reader. Cell viability was calculated relative to the corresponding control group.

### 2.11 Enzyme-linked immunosorbent assay

The concentration of soluble ULBP1 (sULBP1) in cell culture supernatants was measured using a human ULBP1 ELISA kit according to the manufacturer’s instructions. Briefly, culture supernatants were collected from si-NC, si-ULBP1-3, and si-ULBP1-3+OE-ULBP1 MCF-7 cells, centrifuged to remove cell debris, and then subjected to ELISA detection. The absorbance was measured using a microplate reader, and sULBP1 concentrations were calculated based on the standard curve and expressed as pg/mL.

### 2.12 Transwell migration and invasion assays

Transwell assays were performed to evaluate cell migration and invasion abilities (29). MCF-7 cells transfected with si-NC or si-ULBP1 (2.5 × 10^5^ cells/mL) were seeded into the upper chambers containing serum-free medium, while the lower chambers were filled with complete medium containing 10% FBS. After incubation at 37 °C with 5% CO_2_ for 48 h, cells that migrated to the lower surface were fixed with 4% paraformaldehyde (AR, Tianjin Damao Chemical Reagent) for 30 min and stained with 0.1% crystal violet (Q/12GF, Tianjin Guangfu Chemical) for 1 h. Migrated cells were observed and imaged under an optical microscope. For invasion assays, the procedure was identical except that the upper chambers were pre-coated with Matrigel matrix (356234, Corning).

### 2.13 Quantitative real-time PCR

Total RNA was extracted using TriQuick Reagent (R1100, Solarbio), and complementary DNA (cDNA) was synthesized using the Evo M-MLV RT Premix kit (AG11706-S, Accurate Biotechnology). Quantitative PCR was performed using 2× SuperStar Universal SYBR Master Mix (CW3360, CWBIO) on a real-time PCR system. GAPDH was used as an internal control, and relative gene expression levels were calculated using the 2^^−ΔΔCt^ method (30). The primer sequences are provided in **Supplementary Table 1**.

### 2.14 Western blot analysis

Total protein was extracted using RIPA lysis buffer (R0010–100 mL, Solarbio) and quantified with a BCA protein assay kit (CW0014S, CWBIO). Equal amounts of protein were separated by SDS-PAGE and transferred onto PVDF membranes. The membranes were blocked with 5% non-fat milk in TBST at 25 °C for 1 h, and subsequently incubated with primary antibodies against ULBP1 (17715-1-AP, Proteintech) and GAPDH (60004-1-Ig, Proteintech) at 4 °C for 12–16 h. After three washes with TBST, the membranes were incubated with goat anti-rabbit IgG H&L secondary antibody (ZB-2301-0.1 mL, ZSGB-BIO) at 25 °C for 1 h. Protein signals were detected using BeyoECL Moon reagent (P0018FS, Beyotime) and visualized with a Chemi6000 imaging system. Band intensities were quantified using ImageJ software, and ULBP1 protein expression levels were normalized to GAPDH and expressed as the ratio of ULBP1 band intensity to GAPDH band intensity for each sample.

### 2.15 Statistical analysis

All statistical assessments were performed using R version 4.3.3. Discrepancies in expression levels and immune cell abundance were determined by the Wilcoxon test, whereas Pearson’s method was used for correlation studies. Experimental results, displayed as mean ± standard deviation, were analyzed via t-tests (for two groups) or ANOVA (for multiple groups). All tests were two-sided, using a significance threshold of P < 0.05.

## 3 Results

### 3.1 ULBP1 expression in BC and its clinicopathological correlations

**Figure 1:**
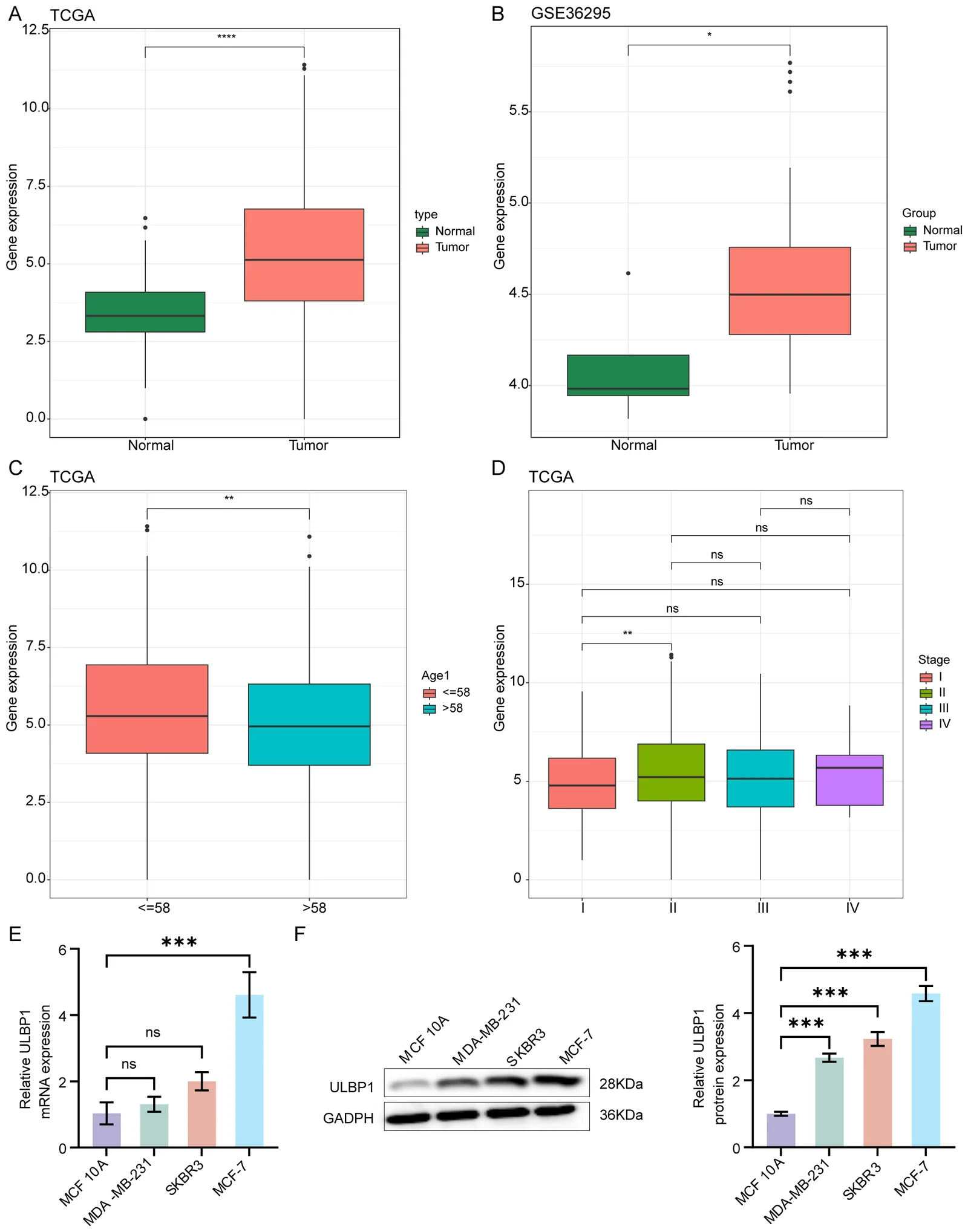
ULBP1 expression in BC tissues and its association with clinicopathological features. **(A)** ULBP1 expression in tumor versus normal breast tissues in the TCGA-BRCA cohort. **(B)** ULBP1 expression in tumor versus normal samples in the GSE36295 cohort. **(C)** ULBP1 expression across age groups in the TCGA-BRCA cohort. **(D)** ULBP1 expression across clinical stage groups in the TCGA-BRCA cohort. **(E)** qRT-PCR analysis of ULBP1 mRNA expression in MDA-MB-231, SKBR3, MCF-7 and MCF-10A. **(F)** Western blot analysis of ULBP1 protein expression in MDA-MB-231, SKBR3, and MCF-7 and MCF-10A. Data are presented as mean ± SD. *P < 0.05, **P < 0.01, ***P < 0.001, ****P < 0.0001, ns, not significant.

ULBP1 expression was compared between breast tumor tissues and normal breast tissues in the TCGA-BRCA cohort. The results clearly demonstrated that ULBP1 was significantly upregulated in tumor samples relative to normal tissues (**Figure 1A**). This finding was further validated in GSE36295, which consistently confirmed a significant increase in ULBP1 expression in BC tissues (**Figure 1B**).

In the TCGA-BRCA cohort, analyses of clinicopathological correlations indicated that ULBP1 expression differed significantly across age groups (**Figure 1C**). Notably, ULBP1 expression showed a significant difference between stage I and stage II cases, whereas no statistically significant differences were observed among the other clinical stage groups (**Figure 1D**). Taken together, these results indicate that ULBP1 is upregulated in BC tissues and is associated with certain clinicopathological characteristics.

Based on the above tissue-level findings, we further examined whether ULBP1 was also differentially expressed in representative BC cell lines. qRT-PCR and western blotting were performed in MCF-10A cells and three BC cell lines, including MCF-7, SKBR3, and MDA-MB-231. Although partial discrepancies between ULBP1 mRNA and protein abundance were observed in some cell lines, which may reflect post-transcriptional, translational, or protein stability-related regulation, MCF-7 cells consistently showed the highest ULBP1 expression at both the mRNA and protein levels (**Figures 1E, F**).

### 3.2 Prognostic value of ULBP1 in BC

**Figure 2:**
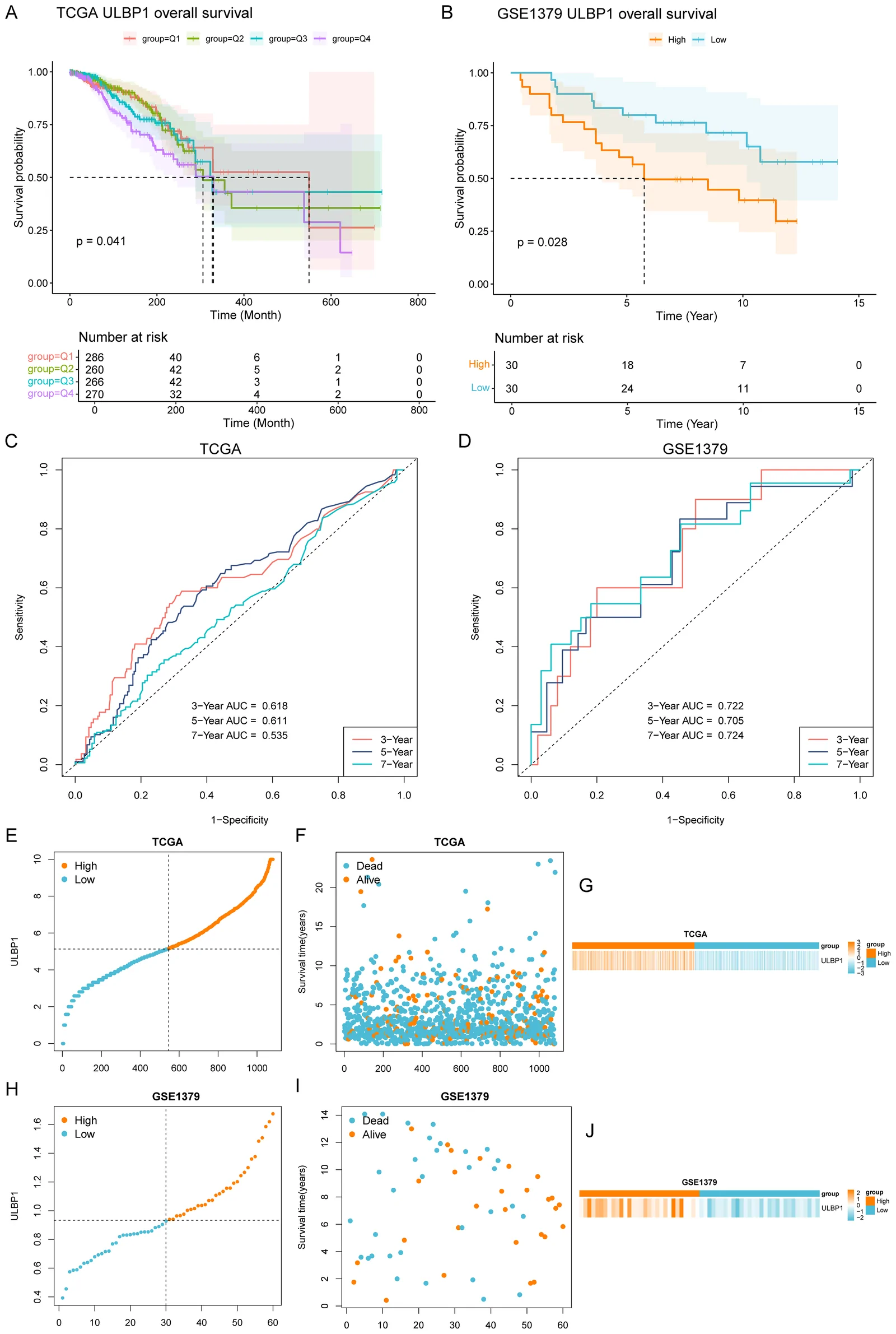
Survival analyses and prognostic prediction of ULBP1. **(A)** Kaplan–Meier OS curves stratified by ULBP1 expression quartiles (Q1–Q4) in the TCGA-BRCA cohort. **(B)** Kaplan–Meier OS curves stratified by the median ULBP1 expression (high vs. low) in the GSE1379 cohort. **(C)** Time-dependent ROC curves (3, 5, and 7 years) for ULBP1 in the TCGA-BRCA cohort. **(D)** Time-dependent ROC curves (3, 5, and 7 years) for ULBP1 in the GSE1379 cohort. **(E–G)** Sample distribution plot, scatter plot of expression versus survival time, and gene expression heatmap based on median ULBP1 stratification in the TCGA-BRCA cohort. **(H–J)** Corresponding plots in the GSE1379 cohort.

To evaluate the prognostic predictive value of ULBP1 in BC, Kaplan–Meier survival analysis was first performed in the TCGA-BRCA cohort. When patients were stratified into quartiles according to ULBP1 expression (Q1–Q4), the high-expression group (Q4) exhibited a significantly poorer OS (**Figure 2A**). To further contextualize these findings within the broader ULBP family, Kaplan-Meier survival analyses were additionally performed for ULBP2 and ULBP3 in the same cohort. ULBP2 high expression was also significantly associated with unfavorable OS (p = 0.043), while ULBP3 showed no significant prognostic value (p = 0.23) (**Supplementary Figures S1A, B**). Notably, ULBP1 demonstrated the most significant association with poor prognosis among the three isoforms examined (p = 0.041), supporting the rationale for focusing on ULBP1 as the primary subject of investigation in the present study. To ensure robustness, an independent K–M analysis was conducted in the GSE1379 cohort, in which patients were dichotomized into high- and low-expression groups using the median ULBP1 expression level. Consistently, elevated ULBP1 expression was associated with worse OS (**Figure 2B**).

Time-dependent ROC curves were subsequently generated in the TCGA-BRCA cohort to assess the predictive accuracy of ULBP1 expression for survival outcomes. ULBP1 showed moderate predictive performance at 3, 5, and 7 years, with AUC values of 0.618, 0.611, and 0.535, respectively (**Figure 2C**). Notably, higher predictive performance was observed in the independent GSE1379 validation cohort, where the 3-, 5-, and 7-year AUC values reached 0.722, 0.705, and 0.724, respectively (**Figure 2D**). To further assess the prognostic performance of ULBP1, the C-index was calculated as an additional validation metric. ULBP1 expression achieved a C-index of 0.606 (95% CI: 0.546–0.666) in the TCGA-BRCA cohort and 0.693 (95% CI: 0.632–0.755) in the GEO cohort (GSE36295) (**Supplementary Figure S1C**), indicating moderate discriminative ability for OS prediction.

Finally, TCGA-BRCA samples were further divided into high- and low-expression groups based on the median ULBP1 level. Visualization analyses, including sample distribution plots, scatter plots of expression versus survival time, and gene expression heatmaps, consistently illustrated a close association between high ULBP1 expression and unfavorable prognosis (shorter survival time) (**Figures 2E–G**). These findings were consistently validated in the GSE1379 cohort (**Figures 2H–J**). Overall, these results indicate that high ULBP1 expression is associated with poorer OS in BC patients and may have potential prognostic value.

### 3.3 Potential prognostic value of ULBP1 and construction of a nomogram model

**Figure 3:**
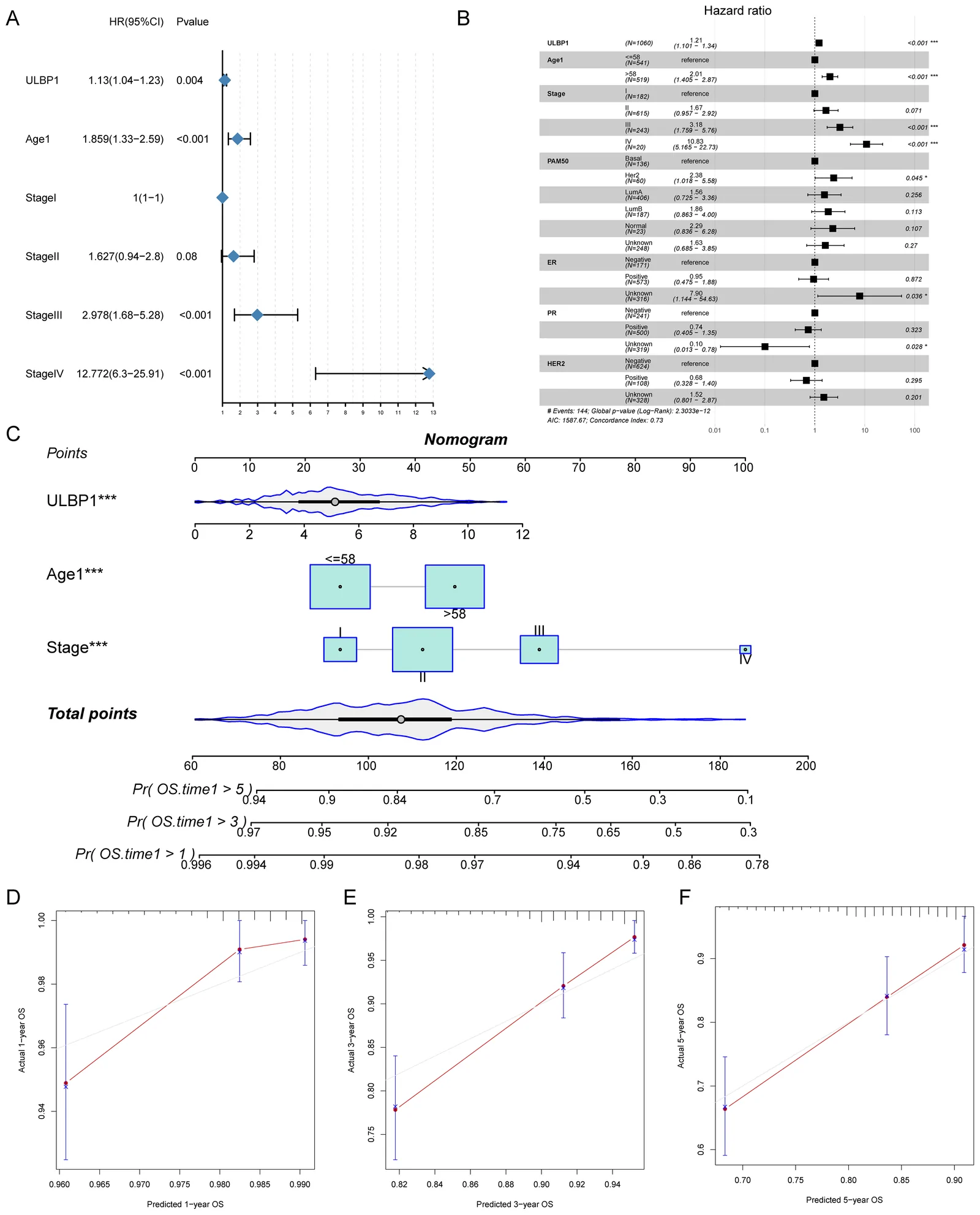
Cox regression analyses and construction of a nomogram model. **(A)** Univariate Cox proportional hazards regression analysis in the TCGA-BRCA cohort including ULBP1 expression, age, and clinical stage (Stage). **(B)** Multivariate Cox proportional hazards regression analysis in the TCGA-BRCA cohort. **(C)** Nomogram integrating ULBP1 expression, age, and stage to predict 1-, 3-, and 5-year OS probabilities. **(D–F)** Calibration curves for the nomogram at 1, 3, and 5 years.

To evaluate the potential prognostic value of ULBP1 in BC, Cox proportional hazards regression analyses were performed based on the TCGA-BRCA cohort. In the univariate Cox regression analysis, ULBP1 expression, age, and clinical stage were incorporated into the model. The results indicated that ULBP1 expression, age, and stage were all significantly associated with OS (P < 0.05) (**Figure 3A**). Subsequently, all variables that reached statistical significance in the univariate analysis were entered into the multivariate Cox regression model. After adjustment for key clinical and molecular covariates, including age, clinical stage, PAM50 subtype, and ER, PR, and HER2 status, ULBP1 expression remained significantly associated with OS in BC patients (P < 0.05) (**Figure 3B**), suggesting that its prognostic value is independent of these established clinical and molecular variables and that ULBP1 may represent a candidate prognostic indicator. To further examine whether the prognostic association of ULBP1 was consistent across different molecular subtype backgrounds, subgroup survival analyses were performed in TNBC and non-TNBC patients. Kaplan–Meier analysis showed that patients with high ULBP1 expression had significantly poorer OS than those with low ULBP1 expression in the TNBC subgroup (**Supplementary Figure S2A**). A similar survival difference was also observed in the non-TNBC subgroup (**Supplementary Figure S2B**). These results suggest that the prognostic relevance of ULBP1 is relatively consistent across TNBC and non-TNBC subgroups, further supporting its robustness as a prognosis-related molecule in BC.

To predict OS probabilities at 1, 3, and 5 years, we developed a prognostic nomogram based on ULBP1 levels and clinical parameters (age and stage) (**Figure 3C**). Validation via calibration curves (**Figures 3D–F**) revealed high concordance between the predicted and actual outcomes, as the curves remained in close alignment with the ideal reference line. These findings underscore the robustness and precision of our model for long-term survival forecasting. Taken together, these results suggest that ULBP1 expression is independently associated with OS prognosis in BC patients and may provide reference value for prognostic risk assessment.

### 3.4 Functional enrichment analysis

**Figure 4:**
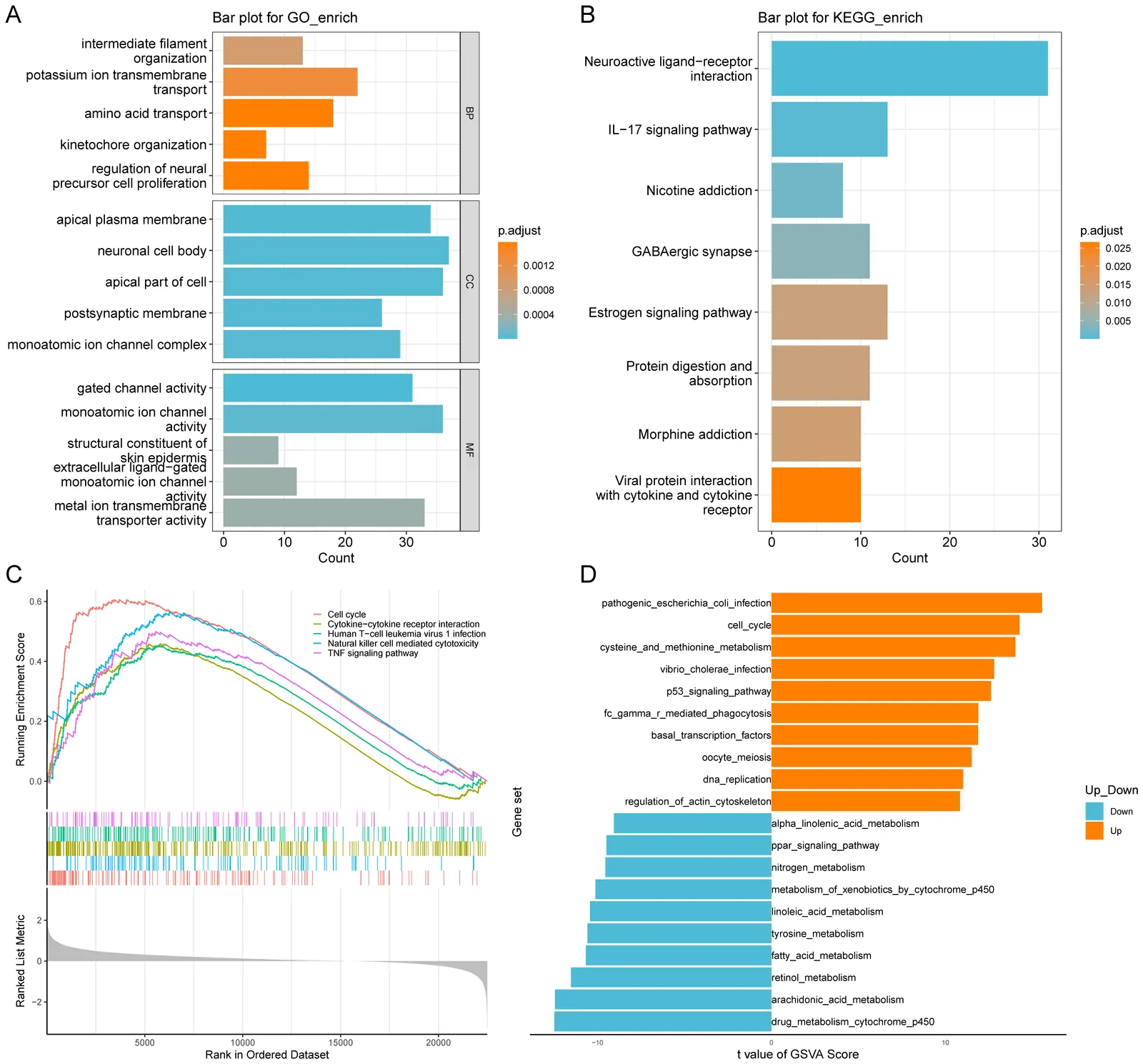
Functional enrichment analyses of DEGs between high- and low-ULBP1 expression groups. **(A)** GO enrichment results for DEGs across BP, MF, and CC categories. **(B)** KEGG pathway enrichment analysis of DEGs. **(C)** GSEA comparing high versus low ULBP1 expression groups. **(D)** GSVA showing differences in KEGG pathway activity.

To further elucidate the potential mechanisms by which ULBP1 may contribute to BC initiation and progression, comprehensive enrichment analyses were performed for DEGs between the high- and low-ULBP1 expression groups. GO enrichment analysis showed that the DEGs were significantly enriched in a total of 252 GO terms, including 130 BP terms (e.g., regulation of neural precursor cell proliferation, kinetochore organization, and amino acid transport), 83 MF terms (e.g., monoatomic ion channel activity, gated channel activity, and structural constituent of skin epidermis), and 39 CC terms (e.g., monoatomic ion channel complex, postsynaptic membrane, and apical part of cell) (**Figure 4A**; **Supplementary Table 2**). In addition, KEGG pathway enrichment analysis identified eight significantly enriched pathways (P < 0.05), including Neuroactive ligand–receptor interaction, IL-17 signaling pathway, and Estrogen signaling pathway (**Figure 4B**; **Supplementary Table 2**).

GSEA further demonstrated that, compared with the low-expression group, the high-expression group exhibited significant enrichment of 34 pathways, including Cell cycle, Cytokine–cytokine receptor interaction, Human T-cell leukemia virus 1 infection, NK cell mediated cytotoxicity, and TNF signaling pathway (**Figure 4C**; **Supplementary Table 2**). Finally, gene set variation analysis (GSVA) was conducted to further confirm differences in pathway activity. The results revealed significant differences between the high- and low-expression groups across 149 KEGG pathways, including DNA replication, Regulation of actin cytoskeleton, and Fc gamma R-mediated phagocytosis (**Figure 4D**; **Supplementary Table 2**). In conclusion, these enrichment analyses indicate that ULBP1 expression is closely associated with alterations in multiple biological processes and immune-related pathways.

### 3.5 Immune infiltration analysis

**Figure 5:**
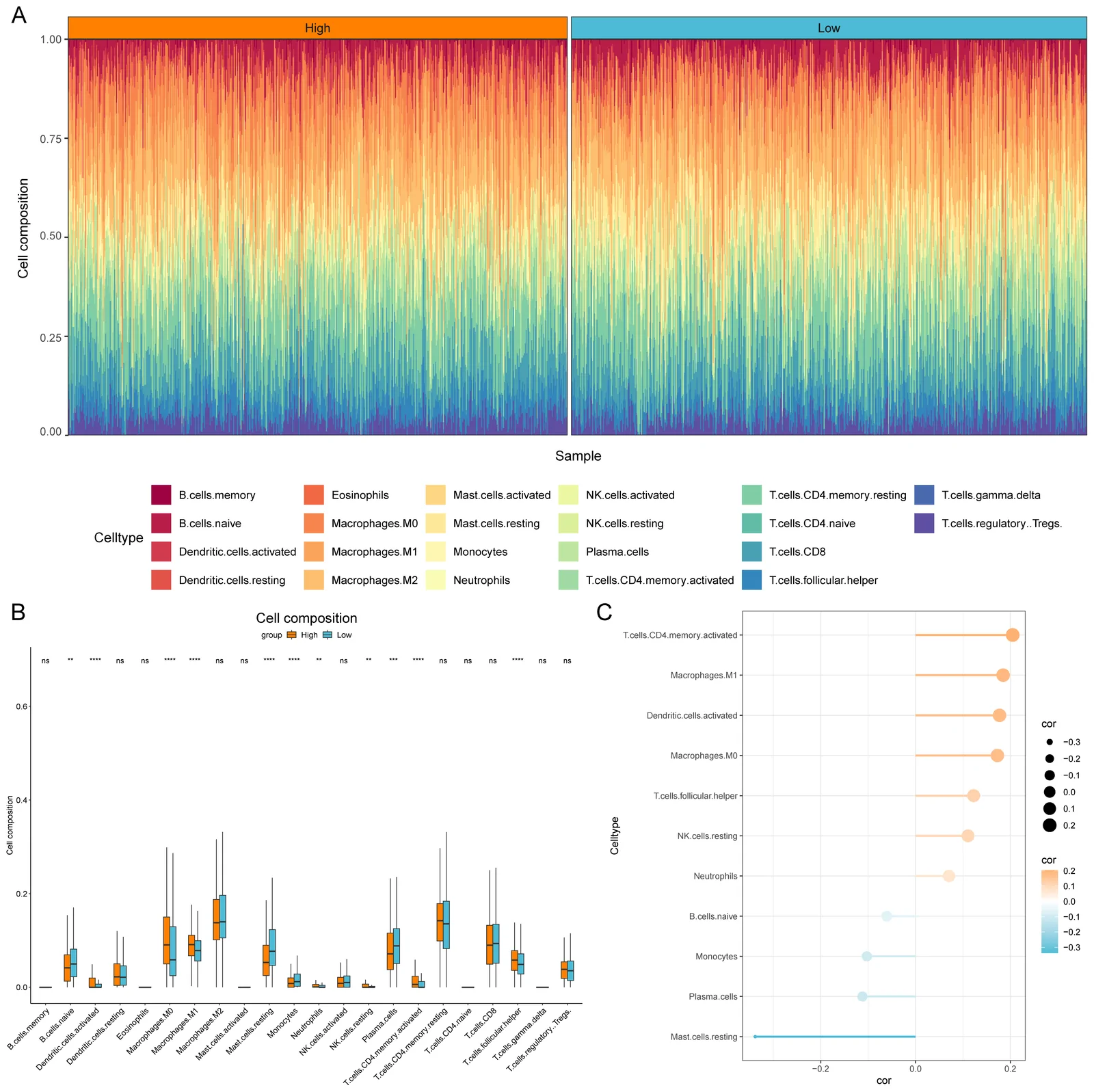
Association between ULBP1 and the TIME in BC. **(A)** Relative proportions of 22 immune cell subtypes in TCGA-BRCA tumor samples estimated by the CIBERSORT algorithm. **(B)** Comparison of immune cell infiltration between high- and low-ULBP1 expression groups. **(C)** Pearson correlation analysis between ULBP1 expression and immune cell infiltration fractions. **P < 0.01, ***P < 0.001, ****P < 0.0001, ns, not significant.

To investigate how high ULBP1 expression influences the tumor immune microenvironment (TIME) in BC, the CIBERSORT algorithm was applied to quantify the relative proportions of 22 immune cell subtypes in tumor samples from the TCGA-BRCA cohort (**Figure 5A**). Subsequently, immune infiltration patterns were compared between the high- and low-ULBP1 expression groups. The results showed that the infiltration levels of 11 immune cell subtypes differed significantly between the two groups. Specifically, activated dendritic cells, macrophages M0, macrophages M1, resting NK cells, follicular helper T cells, activated CD4 memory T cells, and neutrophils were significantly enriched in the high-ULBP1 expression group, whereas naive B cells, monocytes, plasma cells, and resting mast cells were significantly enriched in the low-expression group (**Figure 5B**). To further validate the robustness of these findings, an independent deconvolution analysis was performed using the xCell algorithm, which is methodologically distinct from CIBERSORT in its gene set enrichment-based scoring strategy. The xCell results were consistent with those obtained by CIBERSORT, with the high-ULBP1 expression group similarly exhibiting a cellular composition characteristic of an immunosuppressive phenotype (**Supplementary Figure S3**).

Further Pearson correlation analysis revealed close associations between ULBP1 expression and multiple immune cell populations. ULBP1 expression was significantly positively correlated with the infiltration of activated CD4 memory T cells, macrophages M0, macrophages M1, and resting NK cells, while showing significant negative correlations with naive B cells, monocytes, plasma cells, and resting mast cells (**Figure 5C**). Taken together, these results suggest that ULBP1 expression is closely associated with immune infiltration characteristics in BC and may reflect its potential involvement in the TIME.

### 3.6 ULBP1 expression is associated with predicted immunotherapy response and immune checkpoint gene expression

**Figure 6:**
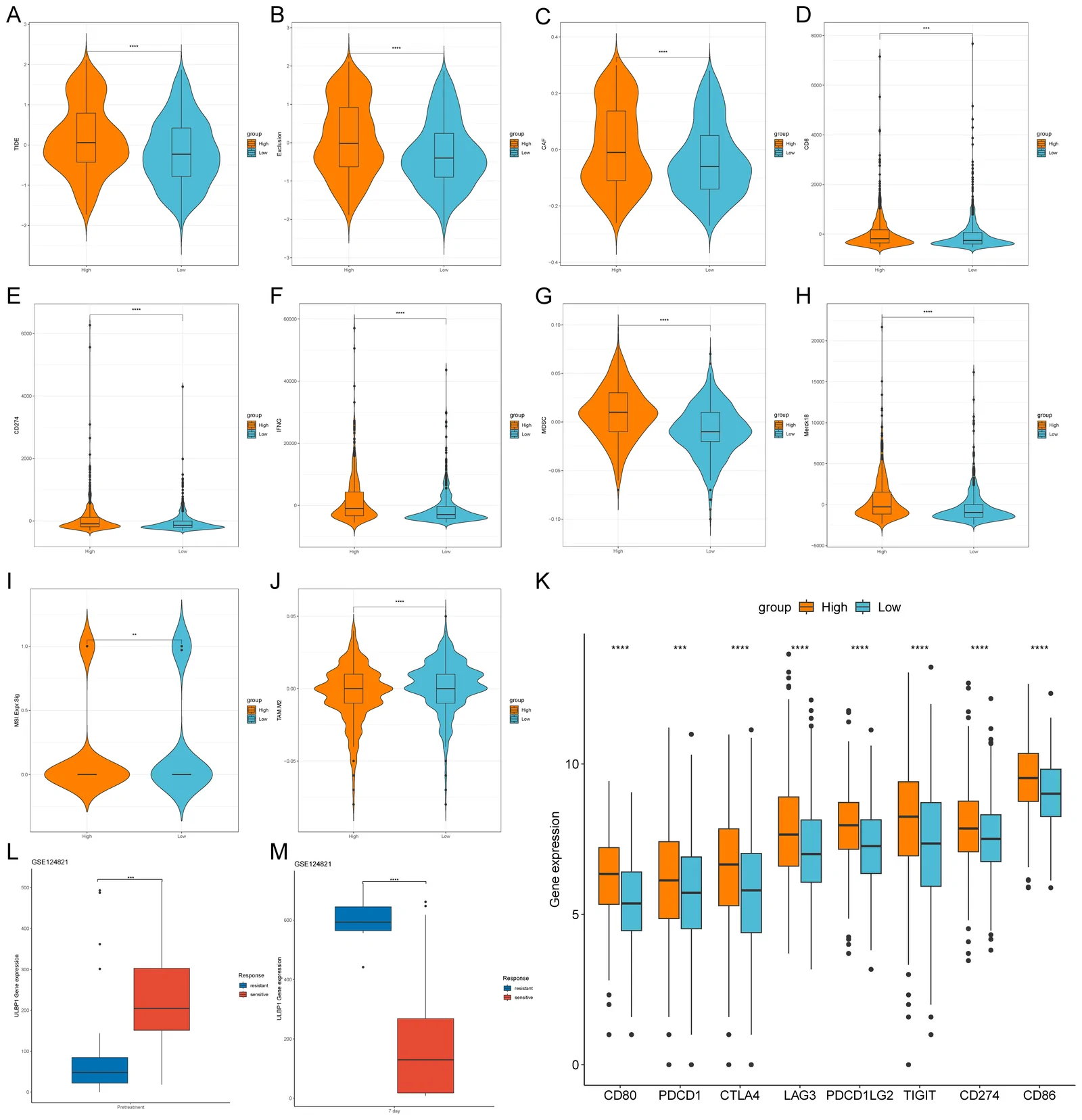
Correlation analysis of ULBP1 expression with TIDE scores, immune checkpoint gene expression, and ICB treatment sensitivity. **(A, B)** TIDE score, Exclusion score between high- and low-ULBP1 expression groups. **(C–J)** Differences in key immune features and cell populations, including CAF, CD8, CD274, IFNG, MDSC, Merck18, MSI.Expr.Sig, and TAM.M2, were analyzed across the two groups. **(K)** Expression comparison of eight immune checkpoint genes between high- and low-ULBP1 groups. **(L, M)** ULBP1 expression in the external TNBC mouse model transcriptomic dataset GSE124821 stratified by ICB sensitivity/resistance at pretreatment and after 7 days of treatment. P < 0.01, P < 0.001, ***P < 0.0001.

The TIDE framework was used to evaluate the association between ULBP1 expression and the response to immune checkpoint inhibitors (ICIs). The results showed that the high-ULBP1 expression group had significantly higher TIDE scores, exclusion scores, and levels of CAF, IFNG, and MSI.Expr.Sig than the low-expression group (**Figures 6A–J**; **Supplementary Table 3**), suggesting that high ULBP1 expression may be potentially associated with reduced benefit from immunotherapy. In parallel, differential expression of eight immune checkpoint genes (CD274, PDCD1, CTLA4, CD80, CD86, LAG3, PDCD1LG2, and TIGIT) was examined between the high- and low-ULBP1 groups. The expression levels of all eight immune checkpoint genes were significantly increased in the high-expression group (**Figure 6K**). To further explore this association in a human ICI-treated context, we analyzed the GSE93157 dataset, which includes RNA-seq data from patients with non-small cell lung cancer, head and neck squamous cell carcinoma, and melanoma before and after anti-PD-1 treatment, together with annotated response information. ULBP1 expression was significantly higher in the non-response group than in the response group (**Supplementary Figure S4**), suggesting that elevated ULBP1 expression may be associated with poorer anti-PD-1 treatment response in a human ICI-treated cohort.

Given that the TIDE analysis suggested that high ULBP1 expression may be associated with limited benefit from ICIs, additional validation was performed using an external transcriptomic dataset from a TNBC mouse model with clearly defined ICB sensitivity/resistance stratification (GSE124821). The results demonstrated that ULBP1 expression was significantly higher in the sensitive group than in the resistant group prior to the initiation of treatment. However, by day 7 of treatment, a dramatic reversal in the expression patterns was observed between the two groups: ULBP1 expression levels surged significantly in the resistant group, whereas a marked decline was noted in the sensitive group (**Figures 6L, M**), suggesting that dynamic changes in ULBP1 expression may be associated with ICB resistance during treatment.

### 3.7 Effects of ULBP1 knockdown on the malignant phenotypes of BC cells

**Figure 7:**
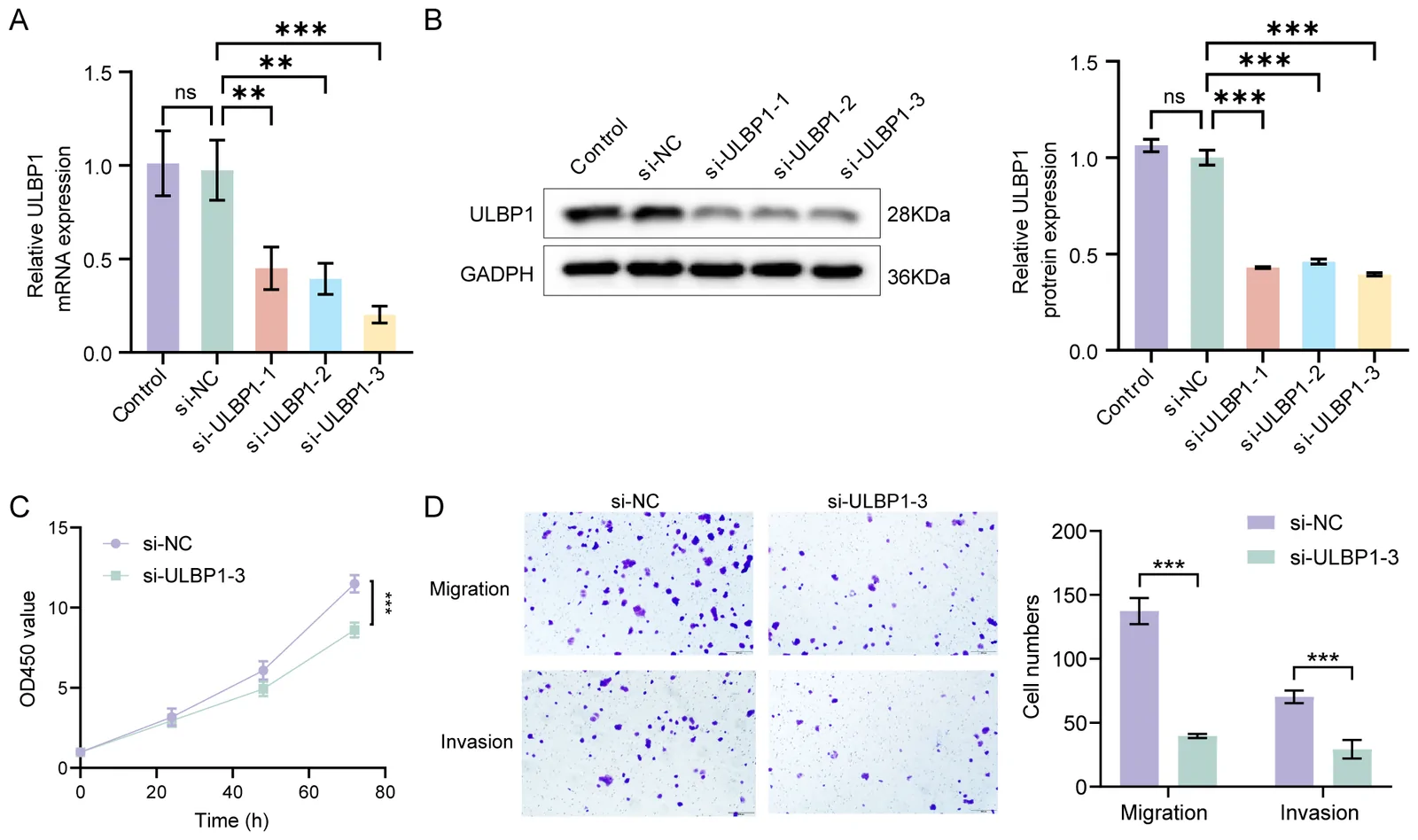
ULBP1 expression in BC cell lines and phenotypic effects of ULBP1 knockdown. **(A)** qRT-PCR analysis of ULBP1 mRNA expression in MCF-7 cells treated with control, si-NC, si-ULBP1-1, si-ULBP1-2, or si-ULBP1-3. **(B)** Western blot analysis of ULBP1 protein expression in MCF-7 cells treated with control, si-NC, si-ULBP1-1, si-ULBP1-2, or si-ULBP1-3. **(C)** CCK-8 assay evaluating the effect of si-ULBP1–3 treatments on MCF-7 cell viability. **(D)** Transwell assay detecting migration and invasion in MCF-7 cells following transfection with si-NC and si-ULBP1-3. Data are presented as mean ± SD. **P < 0.01, ***P < 0.001, ns, not significant.

Based on the validation of ULBP1 expression in BC cell lines, MCF-7 cells exhibited the highest ULBP1 expression among the examined cell lines and were therefore selected as the primary cell model for subsequent loss-of-function experiments. Three ULBP1-targeting siRNAs were designed and synthesized, and their knockdown efficiency in MCF-7 cells was validated by qRT-PCR and Western blot. All three siRNAs effectively reduced ULBP1 expression, among which si-ULBP1–3 achieved the highest silencing efficiency at both mRNA and protein levels (**Figures 7A, B**) and was thus selected for subsequent experiments. Functional analyses demonstrated that effective ULBP1 knockdown significantly suppressed cell viability, as well as cell invasion and migration capacities in MCF-7 cells (**Figures 7C, D**). To assess the generalizability of these findings across BC subtypes, the effect of si-ULBP1–3 on cell viability was further evaluated in the TNBC cell line MDA-MB-231 and the HER2-positive cell line SKBR3. Consistent with the results observed in MCF-7 cells, ULBP1 knockdown significantly reduced cell viability in both MDA-MB-231 and SKBR3 cells (**Supplementary Figure S5**).

**Figure 8:**
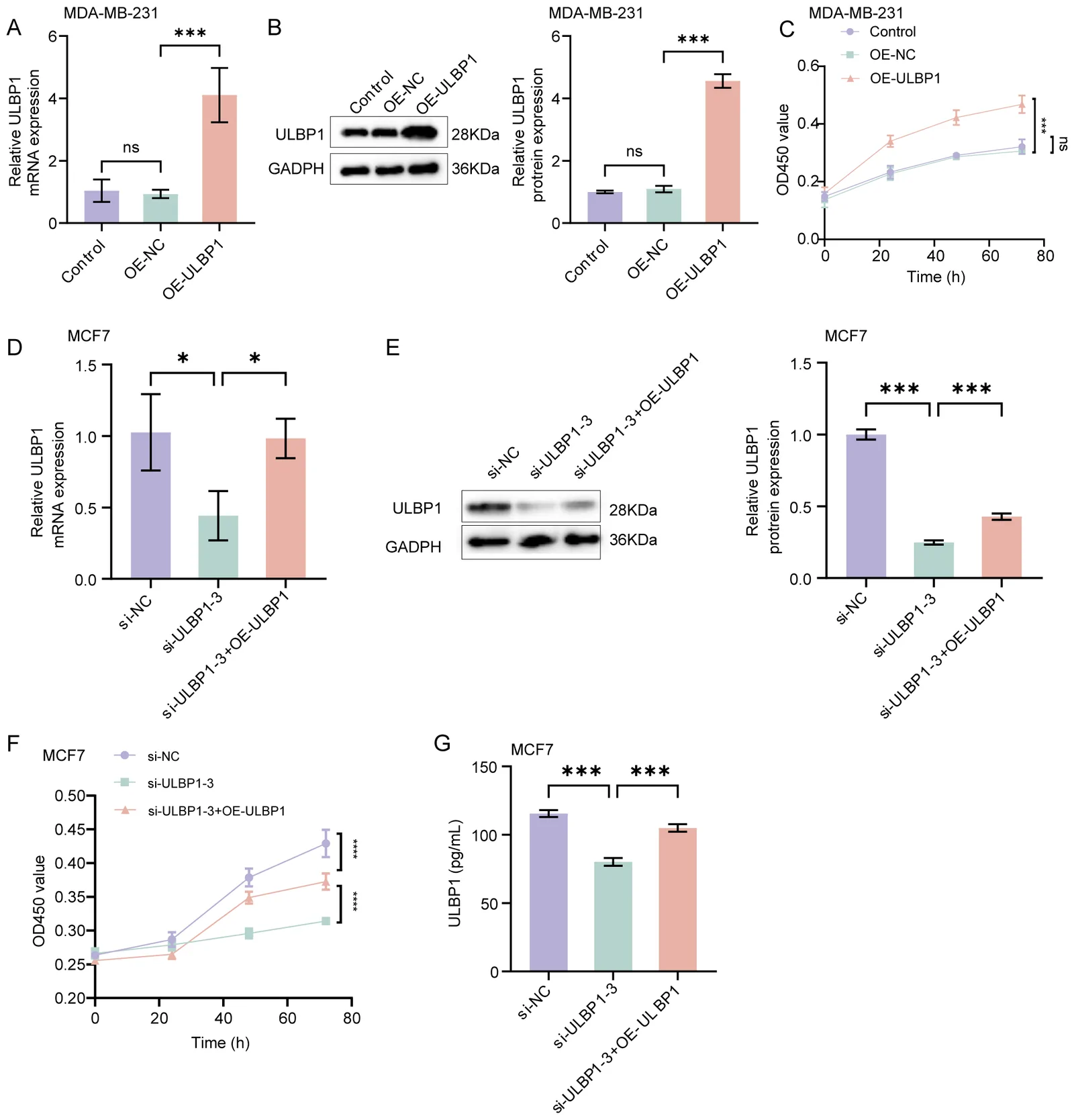
Validation of ULBP1 overexpression in MDA-MB-231 cells, rescue experiments in MCF-7 cells, and detection of sULBP1. **(A)** qRT-PCR analysis of ULBP1 mRNA expression levels in OE-NC and OE-ULBP1 MDA-MB-231 cells. **(B)** Western blot analysis of ULBP1 protein expression levels in OE-NC and OE-ULBP1 MDA-MB-231 cells, with GAPDH used as the loading control. **(C)** CCK-8 assay assessing the effect of ULBP1 overexpression on MDA-MB-231 cell viability. **(D)** qRT-PCR analysis of ULBP1 mRNA expression levels in si-NC, si-ULBP1-3, and si-ULBP1-3+OE-ULBP1 MCF-7 cells. **(E)** Western blot analysis of ULBP1 protein expression levels in si-NC, si-ULBP1-3, and si-ULBP1-3+OE-ULBP1 MCF-7 cells, with GAPDH serving as the internal reference protein. **(F)** Cell viability (OD450) of si-NC, si-ULBP1-3, and si-ULBP1-3+OE-ULBP1 MCF-7 cells at each time point as assessed by CCK-8 assay. **(G)** Concentration of sULBP1 (pg/mL) in the cell culture supernatants of si-NC, si-ULBP1-3, and si-ULBP1-3+OE-ULBP1 groups as quantified by ELISA. Data are presented as mean ± SD. p < 0.05; **p < 0.001. ns, not significant.

To complement the loss-of-function findings, gain-of-function experiments were performed in MDA-MB-231 cells, which exhibited the lowest basal ULBP1 expression among the cell lines examined. Ectopic overexpression of ULBP1 in MDA-MB-231 cells was confirmed at both the mRNA and protein levels (**Figures 8A, B**), and overexpression significantly enhanced cell viability compared with the vector control (**Figure 8C**). To further confirm the on-target specificity of the observed phenotypes, rescue experiments were performed by re-expressing ULBP1 in si-ULBP1-3-transfected MCF-7 cells (si-ULBP1-3 + OE-ULBP1). Both mRNA and protein levels of ULBP1 were restored in the rescue group compared with the si-ULBP1–3 group, as confirmed by qRT-PCR and Western blot (**Figures 8D, E**). CCK-8 assays demonstrated that cell viability was significantly recovered in the si-ULBP1-3+OE-ULBP1 group relative to the si-ULBP1–3 group (**Figure 8F**), confirming that the cell viability of ULBP1 knockdown was specifically attributable to ULBP1 suppression rather than off-target effects.

To further provide evidence for the potential involvement of ULBP1 in immune regulation, we measured the level of sULBP1 in cell culture supernatants. It should be noted that the aforementioned CCK-8, migration, and invasion assays were performed in BC cells cultured alone and therefore mainly reflect the effect of ULBP1 on tumor cell-intrinsic malignant phenotypes. In contrast, sULBP1 detection was intended to assess whether BC cells have the capacity to release sULBP1, thereby providing preliminary clues regarding its potential role in immune evasion. ELISA results showed that sULBP1 levels in the culture supernatants were significantly reduced in the si-ULBP1–3 group compared with the si-NC group and were significantly restored in the si-ULBP1-3+OE-ULBP1 rescue group compared with the si-ULBP1–3 group (**Figure 8G**). These findings suggest that BC cells can release detectable levels of sULBP1 and provide preliminary molecular evidence that ULBP1 may participate in tumor immune evasion in a soluble form.

### 3.8 Predicted association between ULBP1 expression and anticancer drug sensitivity

**Figure 9:**
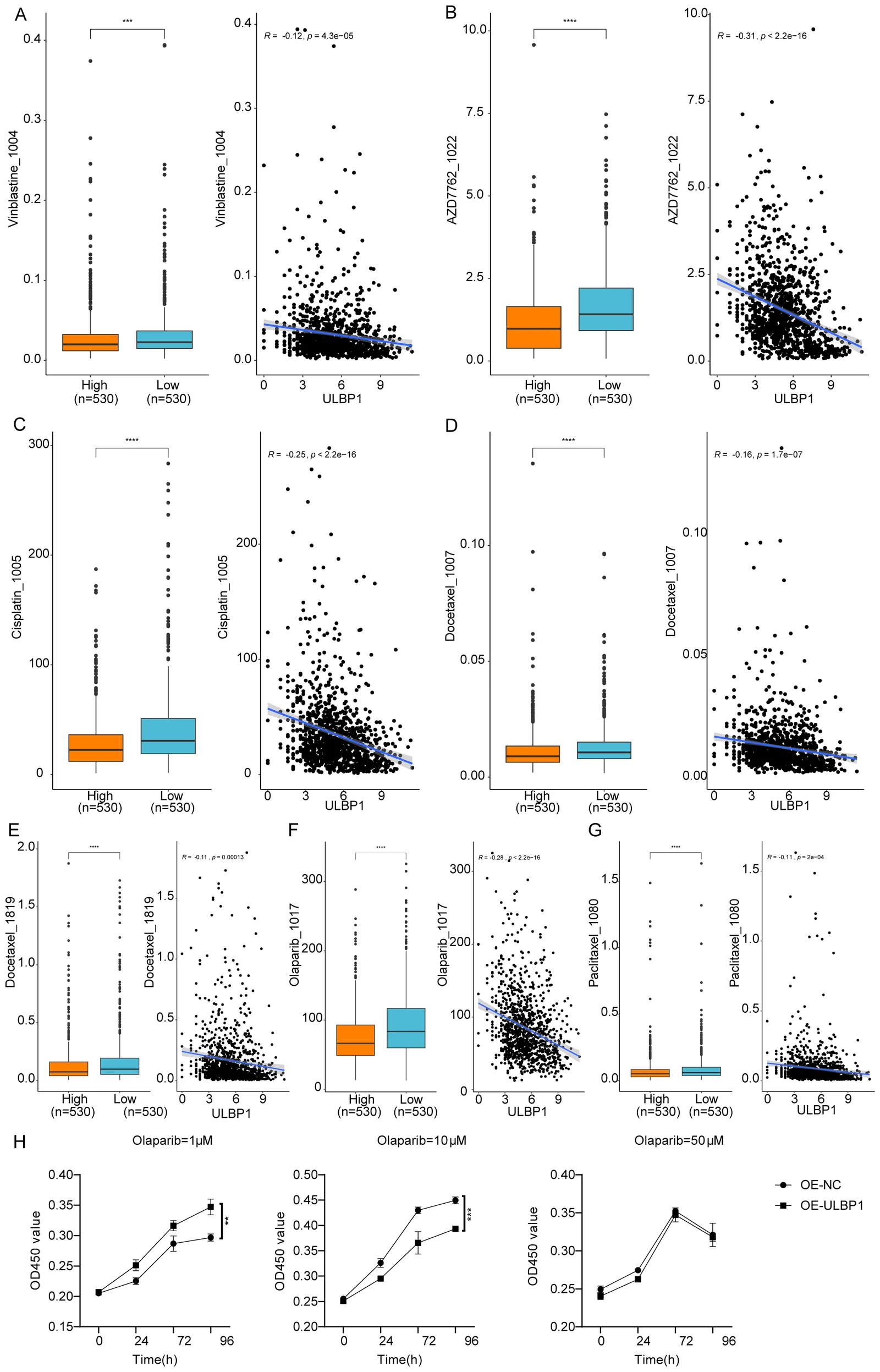
Predicted association between ULBP1 expression and drug sensitivity. **(A)** Vinblastine_1004; **(B)** AZD7762_1022; **(C)** Cisplatin_1005; **(D)** Docetaxel_1007; **(E)** Docetaxel_1819; **(F)** Olaparib_1017; **(G)** Paclitaxel_1080. **(H)** Cell viability (OD450) of OE-NC and OE-ULBP1 MCF-7 cells at 0, 24, 72, and 96 h following treatment with olaparib at concentrations of 1, 10, and 50 μM. Data are presented as mean ± SD. **P < 0.01, ***P < 0.001, ****P < 0.0001.

Using the expression matrix and drug sensitivity data from the GDSC2 database as the training set, the calcPhenotype function in the oncoPredict package was applied to estimate the predicted IC_50_ values of 198 anticancer agents for TCGA-BRCA samples. The association between ULBP1 expression and predicted drug sensitivity was then evaluated by correlating ULBP1 expression levels with the predicted IC_50_ values across the drug panel. To account for multiple comparisons, adjusted p values were calculated, and the corresponding correlation coefficients with confidence intervals are provided in **Supplementary Table 4**. After multiple-testing correction, ULBP1 expression was significantly associated with the predicted IC_50_ values of 136 anticancer drugs. Notably, ULBP1 expression exhibited significant negative correlations with multiple classical chemotherapeutic and targeted agents, including Vinblastine_1004, AZD7762_1022, Cisplatin_1005, Docetaxel_1007, Docetaxel_1819, Olaparib_1017, and Paclitaxel_1080 (**Figures 9A–G**, **Supplementary Table 4**). These findings suggest that BC patients with high ULBP1 expression may be more sensitive to these agents. Among these candidates, olaparib was selected for exploratory *in vitro* validation because ULBP1 expression remained significantly negatively correlated with its predicted IC_50_ value after multiple-testing correction. In addition, olaparib is a clinically relevant PARP inhibitor for selected patients with BC, particularly those with germline BRCA-mutated, HER2-negative disease (31), and its antitumor activity is closely related to DNA damage repair deficiency and synthetic lethality (32). To further evaluate the potential association between ULBP1 and olaparib response, we examined the effects of olaparib on cell viability in ULBP1-overexpressing MCF-7 cells and negative control cells. Under treatment with 1 μM olaparib, cell viability was higher in the OE-ULBP1 group than in the OE-NC group. In contrast, under treatment with 10 μM olaparib, cell viability was significantly lower in OE-ULBP1 cells than in OE-NC cells at the examined time points, suggesting that ULBP1 overexpression may enhance the sensitivity of MCF-7 cells to olaparib at this concentration (**Figure 9H**). At the highest tested concentration of 50 μM, no statistically significant difference in cell viability was observed between the two groups, possibly because the strong cytotoxic effect induced by high-dose olaparib masked ULBP1-dependent differences. These results suggest a potential association between ULBP1 expression and olaparib response in BC cells.

## 4 Discussion

In this study, the expression pattern of ULBP1 in BC and its clinical relevance were evaluated. The results showed that ULBP1 exhibited an upregulated expression trend in BC, and its high expression was significantly associated with unfavorable prognosis. Functional experiments further demonstrated that ULBP1 knockdown markedly suppressed the viability of MCF-7 cells and reduced their migratory and invasive capacities, suggesting that ULBP1 may be involved in maintaining malignant phenotypes in BC. These findings are consistent with previous reports. In BC, NKG2D ligands, including ULBP1, have been frequently detected in tumor tissues by immunohistochemistry, and their expression has been reported to correlate with clinical outcomes (10, 11).

GSEA showed that the “NK cell-mediated cytotoxicity” pathway was significantly enriched in the high-ULBP1 expression group, which is consistent with the classical function of ULBP1 as an NKG2D ligand involved in immune recognition and cytotoxic responses. Under physiological conditions, membrane-bound NKG2D ligands on tumor cells can be recognized by activating receptors expressed on NK cells and cytotoxic T cells, thereby playing a key role in antitumor immunity (33–35). However, in the present study, high ULBP1 expression was significantly associated with unfavorable prognosis. This phenomenon suggests that ULBP1 may have complex functions in the TME beyond its classical immune-activating role. Previous studies have shown that tumor cells can convert membrane-bound ULBP1 into sULBP1 through proteolytic cleavage and other mechanisms. sULBP1 can act as a “molecular decoy” by competitively binding to NKG2D receptors on effector cells and inducing their internalization and degradation, thereby weakening NK cell- and T cell-mediated antitumor immune functions (13, 36, 37). Therefore, we speculate that sULBP1 may play an important role in the apparent contradiction between high ULBP1 expression and poor prognosis. In this context, the function of sULBP1 may be more closely related to immunosuppression than immune activation. Thus, the enrichment of the “NK cell-mediated cytotoxicity” pathway observed in the high-ULBP1 expression group may not necessarily represent effective antitumor immune attack, but may instead reflect an imbalanced state during cancer immunoediting, in which immune activation and immune suppression coexist. Based on these findings, we propose the following hypothesis: in the early stage of tumorigenesis, stress-induced upregulation of membrane-bound ULBP1 may promote NK cell recognition and recruitment, thereby generating a corresponding transcriptomic “activation signature” in the TME. However, under sustained immune selection pressure, tumor cells may achieve immune escape by increasing sULBP1 release, which further leads to impaired or exhausted effector cell function. This transition from “immune clearance” to “immune exhaustion” may generate a paradoxical TME in which effector cells retain partial transcriptional features of activation but have impaired antitumor function. Consistent with this view, previous studies have detected elevated serum levels of soluble NKG2D ligands (sNKG2DLs), including sULBP1, in patients with newly diagnosed BC, and these elevated levels were associated with poor disease prognosis (36). This finding provides a new perspective for understanding the dual role of ULBP1 in BC progression.

Further TME analyses supported an association between high ULBP1 expression and immune microenvironment remodeling. Notably, neutrophils were significantly enriched in the high-ULBP1 expression group. Previous evidence indicates that neutrophils can promote tumor proliferation, angiogenesis, and stromal remodeling by releasing chemokines, reactive oxygen species, and matrix metalloproteinases, while also suppressing T-cell effector functions, thereby facilitating inflammation-driven immune evasion (38, 39). In addition, the TIDE score was significantly higher in the high-ULBP1 group than in the low-expression group, suggesting that patients with high ULBP1 expression may derive limited benefit from immunotherapy. At the molecular level, multiple core immune checkpoint genes, including CD274, PDCD1, CTLA4, LAG3, and TIGIT, were concurrently upregulated in the high-expression group. This pattern typically reflects functional exhaustion of tumor-infiltrating lymphocytes and represents a key hallmark of suppressed antitumor immune responses (40, 41). Collectively, these findings suggest that high ULBP1 expression may correspond to an immunosuppressive microenvironment characterized by neutrophil enrichment and elevated immune checkpoint expression. However, the causal relationship between ULBP1 and the immunosuppressive microenvironment remains to be validated in future mechanistic studies.

In the drug sensitivity prediction analysis, high ULBP1 expression was significantly negatively correlated with the predicted IC_50_ value of olaparib. Olaparib has been approved for the treatment of selected patients with BC, mainly including patients with germline BRCA-mutated, HER2-negative disease in specific advanced/metastatic settings and as adjuvant therapy for high-risk early-stage disease, rather than as a universal therapeutic option for all patients with BC (31, 42–45). To date, the potential association between ULBP1 and olaparib response has not been clearly reported. To further explore the potential association suggested by in silico prediction, we evaluated the effect of olaparib on MCF-7 cells with stable ULBP1 overexpression (OE-ULBP1) and negative control cells (OE-NC). CCK-8 assays showed that ULBP1 overexpression altered the viability response of MCF-7 cells to olaparib in a concentration-dependent manner. Specifically, OE-ULBP1 cells exhibited higher viability than OE-NC cells after treatment with 1 μM olaparib, whereas OE-ULBP1 cells showed significantly lower viability than OE-NC cells under treatment with 10 μM olaparib. At 50 μM olaparib, no significant difference was observed between the two groups, possibly because the strong cytotoxic effect induced by high-dose olaparib masked ULBP1-dependent differences. These findings suggest a potential association between ULBP1 expression and olaparib response in BC cells. However, given that the oncoPredict results were computationally inferred and the experimental validation was performed in a single cell line, this conclusion should be interpreted cautiously. The precise molecular mechanisms by which ULBP1 may influence olaparib response remain to be elucidated and warrant further investigation in future studies.

Several key strengths distinguish the present study from prior work in this area. First, by integrating transcriptomic data from both the TCGA-BRCA cohort and the independent GEO dataset, the expression and prognostic findings reported here are cross-validated across multiple platforms and patient populations, substantially strengthening their reliability. Second, unlike previous studies that examined NKG2DLs primarily from an immunological perspective, the present study adopts a multi-dimensional analytical framework encompassing prognostic modeling, TME deconvolution, immune checkpoint profiling, and drug sensitivity prediction, thereby providing a more comprehensive portrait of ULBP1’s biological significance in BC. Third, the in silico prediction of olaparib sensitivity in ULBP1-high tumors was further corroborated by functional cell-based experiments, in which stable ULBP1 overexpression in MCF-7 cells conferred significantly enhanced sensitivity to olaparib at clinically relevant concentrations.

Despite these strengths, several limitations should be acknowledged. First, a discrepancy was observed between ULBP1 transcript expression patterns in clinical tumor tissues and those in *in vitro* cell lines. Although ULBP1 was significantly upregulated in BC tissues compared with normal breast tissues in both the TCGA-BRCA and GSE36295 cohorts, most BC cell lines showed only modest expression differences relative to MCF-10A, except for MCF-7 cells. This inconsistency may reflect the limitations of conventional cell line models, which do not fully recapitulate the complex TME *in vivo*, including stromal components, immune cell interactions, epigenetic remodeling, and sustained immune selection pressure. Second, the immunotherapy-related analysis requires further validation. Although we performed an exploratory analysis using the human ICI-treated GSE93157 cohort to evaluate the association between ULBP1 expression and anti-PD-1 treatment response, this cohort is not BC-specific and includes multiple tumor types, including non-small cell lung cancer, head and neck squamous cell carcinoma, and melanoma. Therefore, the observed association between high ULBP1 expression and non-response to anti-PD-1 therapy in this cohort should be interpreted as hypothesis-generating evidence rather than direct clinical evidence supporting ULBP1 as a predictive biomarker for ICI response in BC. Further validation in larger BC-specific ICI-treated cohorts is required. Third, the immune microenvironment analysis in the present study was mainly based on computational analyses of public transcriptomic datasets. Although these analyses suggested that high ULBP1 expression was associated with neutrophil-related infiltration signatures and increased immune checkpoint gene expression, these findings remain correlative and computationally inferred. Therefore, the causal relationship between ULBP1 and immune microenvironment remodeling remains to be mechanistically established, and future studies using clinical samples, *in vivo* models, qRT-PCR, immunohistochemistry, flow cytometry, and immune cell co-culture systems are required for further validation. Finally, the signaling pathways through which ULBP1 exerts its tumor-intrinsic effects remain to be clarified. Future studies using primary tumor cells, patient-derived organoids, and *in vivo* models are warranted to better recapitulate the TME and further elucidate the regulatory mechanisms underlying ULBP1 expression and function in BC.

## 5 Conclusion

In conclusion, the present study demonstrates that ULBP1 is upregulated in BC tissues and that its elevated expression is associated with unfavorable prognosis. Functional experiments confirmed that ULBP1 knockdown suppressed the cell viability, migration, and invasion of MCF-7 cells. Additionally, high ULBP1 expression was negatively correlated with predicted olaparib IC_50_ values, and functional validation confirmed that ULBP1 overexpression enhanced olaparib sensitivity in MCF-7 cells. Collectively, these findings identify ULBP1 as a promising prognostic biomarker and potential therapeutic target in BC, warranting further mechanistic and clinical investigation.

## Data Availability

Publicly available datasets were analyzed in this study. This data can be found here: The datasets generated and analysed during the current study are available from The Cancer Genome Atlas (TCGA) database (https://portal.gdc.cancer.gov/) and the Gene Expression Omnibus (GEO) database (https://www.ncbi.nlm.nih.gov/geo/).

## Supplementary material

Supplementary Figure 1 **(A)** Kaplan-Meier survival curve of ULBP2 in the TCGA-BRCA cohort. **(B)** Kaplan-Meier survival curve of ULBP3 in the TCGA-BRCA cohort. **(C)** C-index of ULBP1 in the TCGA-BRCA cohort and the GSE36295 cohort.

Supplementary Figure 2 Subgroup survival analysis of ULBP1 in TNBC and non-TNBC patients. **(A)** Kaplan–Meier survival analysis of ULBP1 expression in the TNBC subgroup. **(B)** Kaplan–Meier survival analysis of ULBP1 expression in the non-TNBC subgroup.

Supplementary Figure 3 xCell-based deconvolution analysis of immune cell infiltration patterns between high- and low-ULBP1 expression groups in the TCGA-BRCA cohort.

Supplementary Figure 4 Association between ULBP1 expression and anti-PD-1 treatment response in the GSE93157 cohort.

Supplementary Figure 5 CCK-8 assay of cell viability in MDA-MB-231 and SKBR3 cells following si-ULBP1–3 transfection.

Supplementary Table 1 siRNA and primer sequence information.

Supplementary Table 2 GO, KEGG, GSEA, and GSVA enrichment analysis results.

Supplementary Table 3 TIDE analysis results.

Supplementary Table 4 Drug sensitivity analysis results.
